# Reverse Transcription Recombinase-Aided Amplification Assay With Lateral Flow Dipstick Assay for Rapid Detection of 2019 Novel Coronavirus

**DOI:** 10.3389/fcimb.2021.613304

**Published:** 2021-02-01

**Authors:** Yu-Zhong Zheng, Jiang-Tao Chen, Jian Li, Xian-Jing Wu, Jin-Zhou Wen, Xiang-Zhi Liu, Li-Yun Lin, Xue-Yan Liang, Hui-Ying Huang, Guang-Cai Zha, Pei-Kui Yang, Lie-Jun Li, Tian-Yu Zhong, Long Liu, Wei-Jia Cheng, Xiao-Nan Song, Min Lin

**Affiliations:** ^1^ School of Food Engineering and Biotechnology, Hanshan Normal University, Chaozhou, China; ^2^ Department of Medical Laboratory, Huizhou Central People’s Hospital, Huizhou, China; ^3^ Department of Human Parasitology, School of Basic Medical Sciences, Hubei University of Medicine, Shiyan, China; ^4^ Department of Medical Laboratory, Center for Disease Control and Prevention, Chaozhou, China; ^5^ Department of Medical Laboratory, Chaozhou People’s Hospital, Shantou University Medical College, Chaozhou, China; ^6^ Department of Research and Development, Chaozhou Hybribio Limited Corporation, Chaozhou, China; ^7^ Department of Laboratory Medicine, First Affiliated Hospital of Gannan Medical University, Ganzhou, China

**Keywords:** Coronavirus Disease-2019, Severe Acute Respiratory Syndrome Coronavirus 2, point of care test****, reverse-transcription recombinase-aided amplification, lateral flow dipstick

## Abstract

**Background:**

The emerging Coronavirus Disease-2019 (COVID-19) has challenged the public health globally. With the increasing requirement of detection for SARS-CoV-2 outside of the laboratory setting, a rapid and precise Point of Care Test (POCT) is urgently needed.

**Methods:**

Targeting the nucleocapsid (N) gene of SARS-CoV-2, specific primers, and probes for reverse transcription recombinase-aided amplification coupled with lateral flow dipstick (RT-RAA/LFD) platform were designed. For specificity evaluation, it was tested with human coronaviruses, human influenza A virus, influenza B viruses, respiratory syncytial virus, and hepatitis B virus, respectively. For sensitivity assay, it was estimated by templates of recombinant plasmid and pseudovirus of SARS-CoV-2 RNA. For clinical assessment, 100 clinical samples (13 positive and 87 negatives for SARS-CoV-2) were tested *via* quantitative reverse transcription PCR (RT-qPCR) and RT-RAA/LFD, respectively.

**Results:**

The limit of detection was 1 copies/μl in RT-RAA/LFD assay, which could be conducted within 30 min at 39°C, without any cross-reaction with other human coronaviruses and clinical respiratory pathogens. Compared with RT-qPCR, the established POCT assay offered 100% specificity and 100% sensitivity in the detection of clinical samples.

**Conclusion:**

This work provides a convenient POCT tool for rapid screening, diagnosis, and monitoring of suspected patients in SARS-CoV-2 endemic areas.

## Introduction

Since early December in 2019, a novel coronavirus (SARS-CoV-2) was firstly detected and reported in Wuhan, China, and it subsequently spread around the world, which presents a major challenge to the global public health community to overcome this novel infectious disease (Coronavirus Disease 2019, COVID-19) (Wang C. et al., 2020). Patients with COVID-19 at early infection exhibit clinical symptoms containing fever, dry cough, and tiredness, and some might experience aches and pains, nasal congestion, sore throat, or diarrhea. It is the light symptoms that cause infected patients not to be aware that they need to be isolated and tested. Furthermore, several symptoms of COVID-19 are flu-like and can easily be misdiagnosed or missed diagnosis. Most people (approximately 80%) recovered from the disease without requiring hospital treatment, while approximately 20% of people infected with COVID-19 become seriously ill and develop difficulty breathing (Huang C. et al., 2020). Some people with low immunity such as the older people and those who are suffering from high blood pressure, heart and lung problems, diabetes, or cancer, are at higher risk of worsening conditions (Huang C. et al., 2020). As of September 7, 2020, the total number of COVID-19 patients is 27 million which is reported in 213 countries and territories around the world. Of these, seven million are currently suffering from COVID-19, and more than 888,727 deaths are caused by COVID-19. Furthermore, about two hundred thousand new COVID-19 patients are being added daily. If the SARS-CoV-2 cannot be effectively contained before the arrival of the winter flu, the global pressure on COVID-19 prevention and control will increase further. It will seriously affect the population’s health and delay economic and social development globally. There is currently no licensed vaccine and no specific antiviral therapy for COVID-19. Therefore, to control the sources of infection and help patients to prevent illness progression, it is essential to provide a reliable, efficient method that can rapidly and accurately identify SARS-CoV-2 RNA.

According to the traditional Koch’s postulates, virus isolation is the “gold standard” for virus diagnosis in the laboratory (Wu et al., 2020). However, virus isolation is not a suitable approach for large-scale detection. Viral nucleic acids can also be used for early diagnosis, which is now widely used in the clinic (Wu et al., 2020). The current reference method for SARS-CoV-2 detection is specific, quantitative real-time polymerase chain reaction (RT-qPCR) testing samples from a nasal or pharyngeal swab, sputum, or bronchoalveolar lavage specimen (Corman et al., 2020; Pfefferle et al., 2020). However, RT-qPCR is costly, more time consuming than RAA, and requires significant laboratory infrastructure and training, hampering its current use in the field, where there are resource limitations (Behrmann et al., 2020; Huang W. E. et al., 2020). Additionally, a lengthy process increases the risk of the potential further spread of SARS-CoV-2 and hinders widespread testing of all potential contacts. To overcome these obstacles, loop-mediated isothermal amplification (LAMP), CRISPR-based fluorescent application, and recombinase polymerase amplification (RPA) have been successfully used in the field to diagnose SARS-CoV-2 infection (Huang W. E. et al., 2020; Huang Z. et al., 2020; Kashir and Yaqinuddin, 2020; Xia and Chen, 2020).

As an isothermal DNA amplification technology, recombinase-aided amplification (RAA) can be used in the field due to its low resource requirements (Fan et al., 2020; Wang Y. et al., 2020).

RAA is a new technology for nucleic acid amplification, and it works using four enzymes (UvsX, UvsY, SSB, and polymerase) at a constant temperature (Zhang et al., 2017). The principle is as follows: UvsX, UvsY, and oligonucleotide primers are combined into complex and then search for the homologous sequences; once it is found, the homologous sequence was displaced as a single-stranded DNA, and the complementary sequence was combined with primers. After that, SSB bound to single-stranded DNA and polymerase bound to the primer–DNA complex, then the amplification was activated. The RAA requires only a simple thermostatic device (constant temperature 37–42°C) and a short reaction time (30 min). DNA amplification can be detected by gel electrophoresis, oligo chromatographic lateral flow dipstick (LFD) (Bai et al., 2020; Xiong et al., 2020), or real-time fluorescence methods (Wang J. et al., 2020; Wang W. et al., 2020), considered as a powerful detection means for Point of Care Test (POCT). Here, we developed a molecular POCT method for SARS-CoV-2 based on a reverse transcription RAA assay coupled with lateral flow dipstick (RT-RAA/LFD) conducted at 39.0°C for 30 min.

## Materials and Methods

### Samples

The study was approved by the Ethics Committee of Chaozhou People’s Hospital affiliated with Shantou University Medical College and the Ethics Committee of Hanshan Normal University. A total of 100 clinical samples were collected from both suspected COVID-19-patients and control populations at Chaozhou People’s Hospital, Huizhou Central Hospital, and Clinical Laboratory Center of the Chaozhou Hybribio Limited Corporation. Viral RNA was extracted from 300 µl throat swabs using a commercial RNA extraction kit (Chaozhou Hybribio Ltd. Corp., Chaozhou, China) for immediate use or stored at −80°C.

### Primers and Probes Design

The primers and probes used for the RT-RAA/LFD assay were designed within conserved regions of the SARS-CoV-2 nucleocapsid (N) gene according to the principles of RAA primer and probe design. NCBI Primer-BLAST was used to confirm the specificity of the primers and probes. The potential for primer–dimer and hairpin formation was analyzed with online Oligo Evaluator software (http://www.oligoevaluator.com). The primers and probe were synthesized by Shanghai Generay Biotech (Shanghai, China). The oligonucleotide sequences of the RAA primers and probe used in the study are listed in **Table 1**.

**Table 1:** Sequences of the RAA primers and LFD probe used in this work.

Primer/Probe	Sequence (5′–3′)
RAA-SARS-2-FW	CTAACAAAGACGGCATCATATGGGTT
RAA-SARS-2-RV1	FITC- GGCCTTTACCAGACATTTTGCTCTCA
RAA-SARS-2-P	Biotin-CTCTTCTCGTTCCTCATCACGTAGTCGCAAC-THF-GTTCAAGAAATTCA -Phosphorylation

THF, tetrahydrofuran spacer; FITC, Fluorescein isothiocyanate.

### RT-RAA/LFD Assay

The RT-RAA/LFD assay was performed using a commercial RT-RAA kit with nfo (Cat. No. T00R01; Qitian Bio-Tech Co., Ltd., Jiangsu, China) (China Invention Patent No. CN201210328735.8) in a reaction volume of  50 µl. The reaction mixtures contained 25 µl of reaction buffer, 10 µl of extracted template, 6.5 µl of ddH_2_O, 0.6 µl of the probe (10 µM), 2.1 µl of the forward primer (10 µM), 3 µl of the FITC-labeled reverse primer (10 µM), and 2.8 µl of 280 mM magnesium acetate. After all the ingredients have been added to the reaction system, it was shaken repeatedly upside down and mixed well 10 times. The reaction mixture was typically incubated for 30 min at 39°C in Thermo Cell (Cat. No. B6100; Qitian, P.R. China). The amplification product was then diluted 50 times with phosphate-buffered saline and tested using commercial lateral flow dipsticks (rainbow) (Cat. No. JY0201; Tiosbio, Beijing, China). Both the test line and control line appear simultaneously indicating a positive result, while only the control line appears to indicate a negative result. Only a test line appears to indicate a doubtful result and needs to be retested.

### Specificity and Sensitivity of the RT-RAA/LFD Assays

The specificity of the RT-RAA/LFD assays was evaluated using human coronaviruses (HCoV-OC43; HCoV-HKU1; HCoV-NL63; HCoV-229E; SARS-CoV), human influenza A virus (H1N1 and H3N2 subtypes), influenza B viruses (Yamagata and Victoria lineages), and respiratory syncytial virus (RSV, type A and B) and hepatitis B virus (HBV). We obtained these viruses from Chaozhou Hybribio Limited Corporation.

The analytical sensitivity of the RT-RAA/LFD assay was determined using recombinant pUC57 plasmid and SARS-CoV-2 RNA transcribed *in vitro* reference material (pseudovirus), respectively. The recombinant pUC57 plasmid containing the full-length N gene of SARS-CoV-2 (GenBank No. MN908947.3) was synthesized by the company (Sangon Biotec Co., Ltd., Shanghai, China). The reference material (low concentration) containing the full-length N (10^4^ copies/µl) and E (10^4^ copies/µl) gene, and the partial sequence of ORF1ab gene (GenBank No.MT027064.1:1,3321–1,5540) (10^4^ copies/µl) was produced by SIMT (Shanghai Institute of Measurement and Testing Technology), and was approved by State Administration for Market Regulation of People’s Republic of China (Certificate No. GBW(E)091111). The recombinant plasmid was diluted to 10^6^, 10^5^, 10^4^, 10^3^, 10^2^, 10^1^, and 1 copies/µl. The reference material was diluted to 10^4^, 10^3^, 10^2^, 10^1^, and 1 copies/µl. Ribozyme-free water was used as a negative control.

### Evaluation of the RT-RAA/LFD Assay Using Clinical Samples

To evaluate the performance of the RT-RAA/LFD assay for SARS-CoV-2 detection, 100 clinical samples were evaluated with a commercial SARS-CoV-2 RT-qPCR kit (Chaozhou Hybribio Ltd.Corp., Chaozhou, PRC) and the RT-RAA/LFD assay. The concordance rate between the two assays was calculated with the following formula: (Number of consistent results by both methods/total number) × 100%.

## Results

### Primer Design for the RT-RAA/LFD Assay

**Figure 1:**
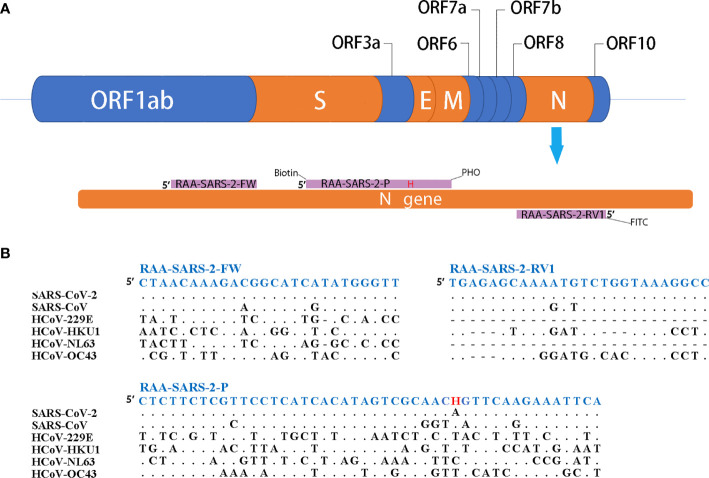
Primers and probe design for RT-RAA/LFD. **(A)** Partial structure of the SARS-CoV-2 gene and locations of primers and probe. H indicates the tetrahydrofuran spacer (THF). Biotin indicates the Biotin label. PHO indicates phosphorylation blocking. FITC indicates the FITC fluorescein label. **(B)** Alignments of designed primers and probes and six coronaviruses. Dots indicate consensus, bars indicate absence.

As shown in **Figure 1**, the primers and probes for the RT-RAA/LFD assay were manually designed, and the alignment among the relative virus strains was presented. We could find that both the SARS-CoV-2 (GenBank: MN908947.3) and SARS-CoV (GenBank: AY274119.3) could be amplified by the pair of primers. Therefore, based on the different regions of the two virus sequences, we designed one specific probe to distinguish them. In theory, the combination of the two primers and one probe could specifically detect SARS-CoV-2. Biotin and fluorescein isothiocyanate (FITC) were labeled at the probe and primer, respectively (**Figure 1**).

### The Analytical Specificity of the RT-RAA/LFD Assay

**Figure 2:**
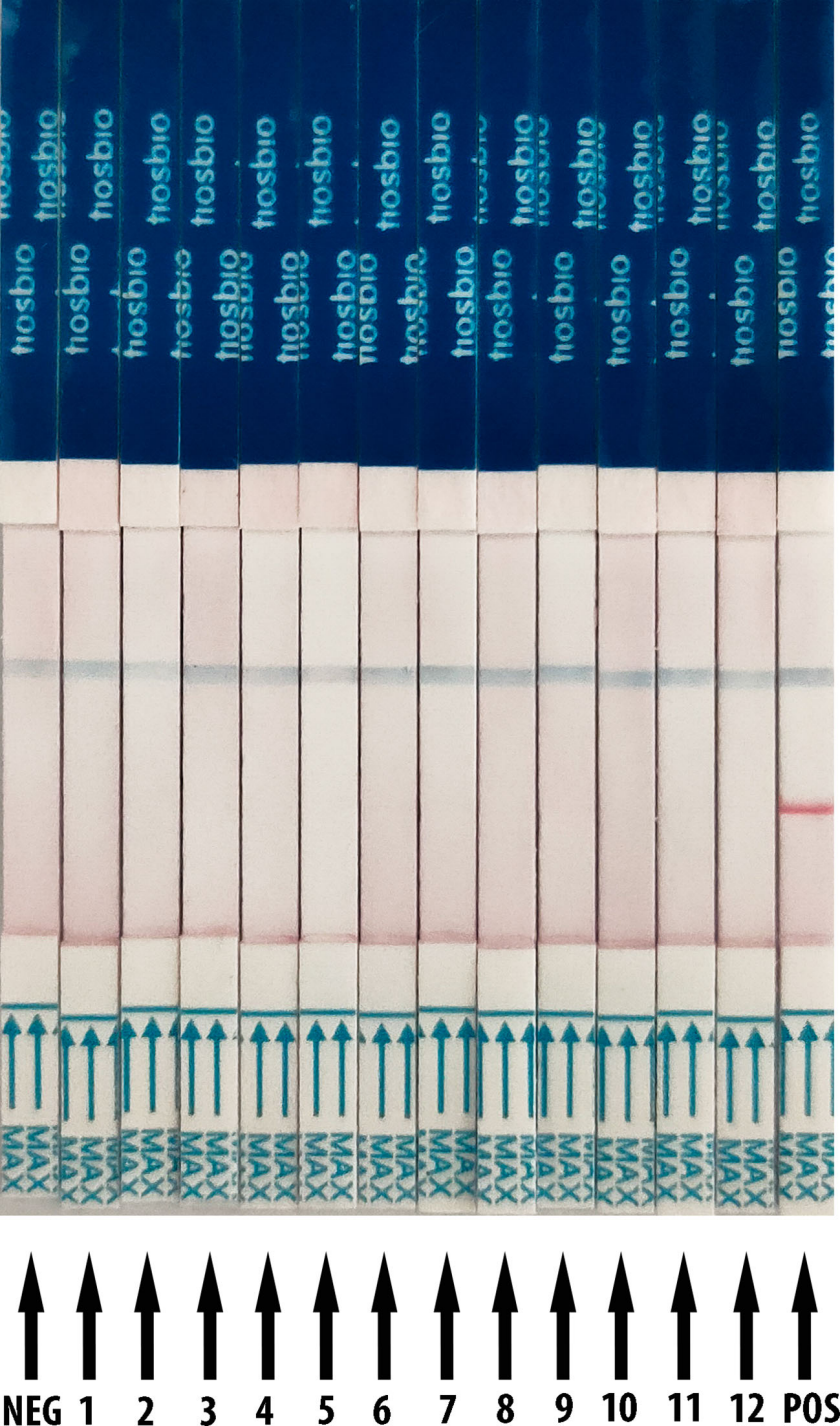
Analytical specificity result of RT-RAA/LFD assay. RT-RAA/LFD specificity result. Blue for the control line while red for the test line. From left to right, the test samples are NEG for ribozyme-free water, 1 for H1N1, 2 for H3N2, 3 for influenza B viruses (Yamagata lineage), 4 for influenza B viruses (Victoria lineage), 5 for respiratory syncytial virus type A, 6 for respiratory syncytial virus type B, 7 for hepatitis B virus, 8 for HCoV-OC43, 9 for HCoV-HKU1, 10 for HCoV-NL63, 11 for HCoV-229E, 12 for SARS-CoV, POS for nucleic acid from SARS-CoV-2.

The RT-RAA reactions were performed with nucleic acid from SARS-CoV-2, human coronaviruses (hCoV), human influenza virus, and respiratory syncytial virus, and HBV as templates. For the LFD detection result (**Figure 2**), it demonstrates only the product of SARS-CoV-2 which showed positive results with both test line and control line, while the products of other relative virus and negative control showed negative results (only quality control line). These results support that the RT-RAA/LFD assay could effectively distinguish SARS-CoV-2 and another viruses. The RT-RAA/LFD assay for the detection of SARS-CoV-2 exhibited high specificity (100%).

### Analytical Sensitivity of RT-RAA/LFD Assay

**Figure 3:**
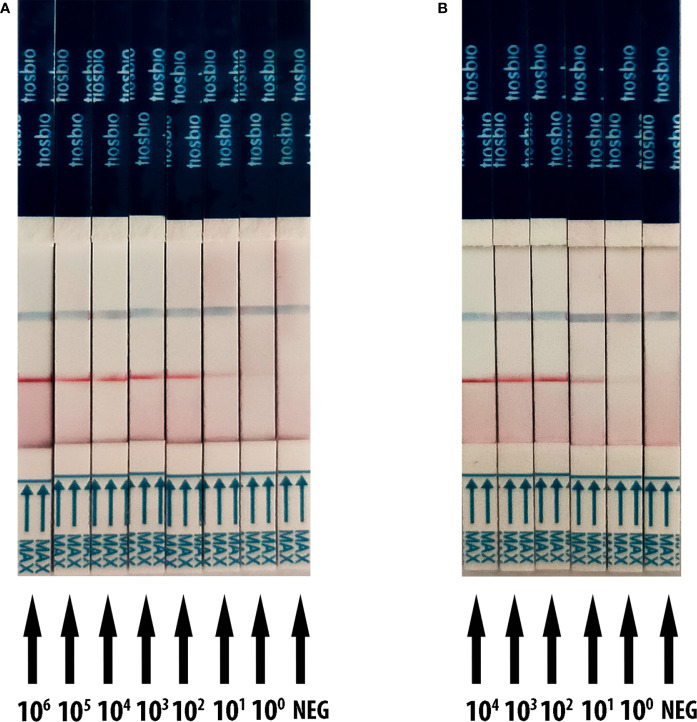
Analytical sensitivity result of RT-RAA/LFD for plasmid and SARS-CoV-2 RNA transcribed *in vitro* reference material (pseudovirus). Blue for the control line while red for the test line. **(A)** The sensitivity results of RT-RAA/LFD for plasmid. 10^6^ to 10^0^ indicates tests with 1 × 10^6^ to 1 copies/µl recombinant plasmid as a template. NEG indicates a test with ribozyme-free water as a template. **(B)** The sensitivity results of RT-RAA/LFD for SARS-CoV-2 RNA transcribed *in vitro* reference material. 10^4^ to 10^0^ indicates tests with 1 × 10^4^ to 1 copies/µl reference material as a template. NEG indicates a test with ribozyme-free water as a template.

The detection threshold of RT-RAA/LFD was determined by using a recombinant plasmid containing the N gene of SARS-CoV-2 and SARS-CoV-2 RNA transcribed *in vitro* reference material (pseudovirus). The dilution series of plasmids ranged from 1 × 10^6^ to 1  copies/µl. For the plasmid, the gradient of the red test band ranged from 1 × 10^6^ to 1  copies/µl was detected (**Figure 3A**). For the SARS-CoV-2 RNA dilution series from 1 ×  10^4^ to 1  copies/µl, we also observed a steadily decreasing trend in the signal from 1 ×  10^4^ to 1 copies/μl (**Figure 3B**). All the results showed that the limit of detection (LOD) was 1 copies/μl for RT-RAA/LFD.

### Clinical Sample Detection *via* the RT-RAA/LFD Assay

To evaluate the clinical performance of the SARS-CoV-2 RT-RAA/LFD assay, RNAs obtained from clinical samples were detected with RT-RAA/LFD and RT-qPCR, respectively. As shown in **Table 2**, the RT-qPCR results illustrated 13 samples were positive for SARS-CoV-2, with CT values ranging from 21.16 to 37.51 (10^5^ to 10^1^ copies/µl). The results of RT-RAA/LFD assays were in complete agreement with those obtained by RT-qPCR. All these results exhibited the excellent performance of RT-RAA/LFD assay applied to detect SARS-CoV-2 in a clinical setting, with a coincidence rate with RT-qPCR results of 100%.

**Table 2:** Comparison of RT-qPCR and RT-RAA/LFD results for clinical samples.

No.	RT-qPCR result (Ct value)			RT-RAA/LFD
	Orf1ab	N-gene	E-gene	Judgment
1	32.97	32.8	31.64	+
2	27.29	26.92	25.89	+
3	33.52	33.75	32.09	+
4	31.23	30.87	29.65	+
5	33.15	33.04	32.10	+
6	35.89	34.60	33.97	+
7	28.04	27.41	26.56	+
8	21.75	21.98	22.15	+
9	27.00	26.44	26.21	+
10	30.78	31.10	30.56	+
11	39.05	37.51	38.10	+
12	34.95	35.27	35.18	+
13	21.38	21.16	20.51	+
Mean	30.54	30.22	29.59	

RT-qPCR and RT-RAA/LFD represent quantitative real-time polymerase chain reaction, reverse-transcription recombinase-aided amplification coupled with lateral flow dipstick, respectively.

## Discussion

The current outbreak of the novel coronavirus COVID-19 (coronavirus disease 2019; the pathogen referred to as SARS-CoV-2; previously 2019-nCoV) has now spread to six continents and had been reported in all but six countries as of 24:00 on September 7, 2020. To control the spread of infectious diseases, including the COVID-19, the most effective strategies are conducting the triple “T” motto: Trace, Test, Treat (Griffin, 2020; Ng et al., 2020). With the awareness of the severe outbreak, the affected countries have taken appropriate measures to conduct extensive detection and effective patients’ screening. Precise POCT methods for the detection of COVID-19 would increase the potential scope of diagnosis and apply in the situations such as the customs, airports, and substrate hospitals that outside of the laboratory settings. Such methods would have the potential to reduce the time required to obtain a reliable result, which could assist in the early identification of COVID-19 infected patients and would also enable the effective use of epidemic prevention and control materials, better development of effective quarantine measures and help with recruitment into clinical trials of treatments.

The earliest available genomic sequence data verified that SARS-CoV-2 is a member of the *Betacoronavirus* genus and belongs to a subgenus (*Sarbecovirus*) that includes SARS-CoV (MERS-CoV belongs to a separate subgenus, *Merbecovirus*) (Lvov and Alkhovsky, 2020; Rabaan et al., 2020). SARS-CoV-2 shows approximately 79% similarity to SARS-CoV at the nucleotide level (Sen et al., 2020). Considering that SARS-CoV was once responsible for outbreaks in mainland China, Hong Kong, Taiwan, Singapore, Vietnam, Canada, and eventually 32 countries or regions (Xie and Chen, 2020) and genomic similarity with the SARS-CoV-2, we designed a specific probe to provide SARS-CoV-2 specific LFD detection.

In this study, the RT-RAA/LFD was completed in 30 min at a constant temperature of 39°C, which represents a significant reduction of the turnaround time compared to other available methods, such as RT-LAMP (50–60 min) (El-Tholoth et al., 2020; Huang W. E. et al., 2020; Kashir and Yaqinuddin, 2020; Yan et al., 2020) and RT-qPCR (2–3 h) (Behrmann et al., 2020; Pfefferle et al., 2020). Our assay shows advantages in terms of sensitivity, timeliness, and simplicity compared to the second detection scheme for SARS-CoV-2 using RAA chemistry developed by Zhang et al. (Wang X. et al., 2020), which is reported to detect 120 RNA copies within 1 h with a simple colorimetric, no-quantitative endpoint readout. In contrast to the dual-gene detection of SARS-CoV-2 RNA *via* a modified RT-RAA approach reported by Chen et al (Xia and Chen, 2020), our assay does not require separate steps to complete reverse transcription and amplification. Furthermore, the analytical sensitivity of our RT-RAA/LFD is 1 copies/μl, which is more sensitive than the fluorescence RT-RAA assay (10 copies/μl) for reported by Wang J. et al. (2020) and Xue et al. (2020). Compared with real-time fluorescence RT-RAA assay (Xue et al., 2020) and RT-qPCR, the RT-RAA/LFD approach does not require a fluorescence detection device and is more suitable for application in resource-poor areas. However, there is a risk of aerosol pollution when using the RT-RAA/LFD method, resulting from the opening of the reactor tubes. Accordingly, RT-RAA/LFD is recommended for application in more device-poor areas or outdoors, while the real-time fluorescence RT-RAA method is recommended for use in conventional testing schemes.

To validate the clinical application of the RT-RAA/LFD strategies, 100 clinical samples were tested. The agreement between the RT-RAA/LFD and RT-qPCR assay was one hundred percent, suggesting that the RT-RAA/LFD assay developed in this study was feasible for screening or diagnosing COVID-19.

In conclusion, the developed POCT method exhibits sufficient specificity and sensitivity and provides a tool for moving forward with rapid screening and diagnosis in community settings (such as primary hospitals, schools, airports, and custom houses), where patients with suspected symptoms could be diagnosed at an early stage. Additionally, we recommend accelerating the development and evaluation of effective molecular POCT systems for SARS-CoV-2.

## Data Availability Statement

The original contributions presented in the study are included in the article/supplementary material. Further inquiries can be directed to the corresponding author.

## Ethics Statement

Written informed consent was obtained from the individuals’ and minors’ legal guardians/next of kin, for the publication of any potentially identifiable images or data included in this article.

## Author Contributions

ML, Y-ZZ, JL, and J-TC designed the experiments. ML, X-JW, J-ZW, X-ZL, LL, W-JC, X-NS, and L-YL developed and evaluated the RT-RAA assay. G-CZ, P-KY, L-JL, and T-YZ performed the experiments. Y-ZZ, J-TC, and ML wrote the manuscript. ML, JL, X-YL, and H-YH edited the manuscript. All authors contributed to the article and approved the submitted version.

## Funding

This study was supported by the Special technology program of Chaozhou for novel coronavirus infection control (Grant/Award Numbers: CZK20200602 and CZK20200603); Special technology program of Guangdong Provincial Education Department for novel coronavirus infection control (Grant/Award Numbers: 2020KZDZX1146); Key scientific research projects of Guangdong Provincial Department of Education (Grant/Award Numbers: 2018A030307074).

## Conflict of Interest

Author L-JL was employed by the company Chaozhou Hybribio Limited Corporation, Chaozhou, Guangdong Province, People’s Republic of China.

The remaining authors declare that the research was conducted in the absence of any commercial or financial relationships that could be construed as a potential conflict of interest.

## References

[B1] Bai X. Ma X. Li M. Li X. Fan G. Zhang R. (2020). Field applicable detection of hepatitis B virus using internal controlled duplex recombinase-aided amplification assay and lateral flow dipstick assay. J. Med. Virol. 10.1002/jmv.25778 32190907

[B2] Behrmann O. Bachmann I. Spiegel M. Schramm M. El Wahed A. A. Dobler G. (2020). Rapid detection of SARS-CoV-2 by low volume real-time single tube reverse transcription recombinase polymerase amplification using an exo probe with an internally linked quencher (exo-IQ). Clin. Chem. 66(8), 1047–1054 10.1093/clinchem/hvaa116 32384153 PMC7239256

[B3] Corman V. M. Landt O. Kaiser M. Molenkamp R. Meijer A. Chu D. K. (2020). Detection of 2019 novel coronaviru -nCoV) by real-time RT-PCR. Euro. Surveill. 25 (3), 2000045. 10.2807/1560-7917.ES.2020.25.3.2000045 PMC6988269 31992387

[B4] El-Tholoth M. Bau H. H. Song J. (2020). A Single and Two-Stage, Closed-Tube, Molecular Test for the 2019 Novel Coronavirus (COVID-19) at Home, Clinic, and Points of Entry. ChemRxiv [Preprint]. 10.26434/chemrxiv.11860137.v1

[B5] Fan X. Li L. Zhao Y. Liu Y. Liu C. Wang Q. (2020). Clinical Validation of Two Recombinase-Based Isothermal Amplification Assays (RPA/RAA) for the Rapid Detection of African Swine Fever Virus. Front. Microbiol. 11, 1696. 10.3389/fmicb.2020.01696 32793160 PMC7385304

[B6] Griffin S. (2020). Covid-19: Test and trace programmes are important but no silver bullet, say scientists. BMJ 369:m2151. 10.1136/bmj.m2151 32467147

[B7] Huang C. Wang Y. Li X. Ren L. Zhao J. Hu Y. (2020). Clinical features of patients infected with 2019 novel coronavirus in Wuhan, China. Lancet 395 (10223), 497–506. 10.1016/S0140-6736(20)30183-5 31986264 PMC7159299

[B8] Huang W. E. Lim B. Hsu C. C. Xiong D. Wu W. Yu Y. (2020). RT-LAMP for rapid diagnosis of coronavirus SARS-CoV-2. Microb. Biotechnol. 13 (4), 950–961. 10.1111/1751-7915.13586 32333644 PMC7264870

[B9] Huang Z. Tian D. Liu Y. Lin Z. Lyon C. J. Lai W. (2020). Ultra-sensitive and high-throughput CRISPR-p owered COVID-19 diagnosis. Biosens. Bioelectron. 164, 112316. 10.1016/j.bios.2020.112316 32553350 PMC7245202

[B10] Kashir J. Yaqinuddin A. (2020). Loop mediated isothermal amplification (LAMP) assays as a rapid diagnostic for COVID-19. Med. Hypotheses 141, 109786. 10.1016/j.mehy.2020.109786 32361529 PMC7182526

[B11] Lvov D. K. Alkhovsky S. V. (2020). [Source of the COVID-19 pandemic: ecology and genetics of coronaviruses (Betacoronavirus: Coronaviridae) SARS-CoV, SARS-CoV-2 (subgenus Sarbecovirus), and MERS-CoV (subgenus Merbecovirus).]. Vopr. Virusol. 65 (2), 62–70. 10.36233/0507-4088-2020-65-2-62-70 32515561

[B12] Ng Y. Li Z. Chua Y. X. Chaw W. L. Zhao Z. Er B. (2020). Evaluation of the Effectiveness of Surveillance and Containment Measures for the First 100 Patients with COVID-19 in Singapore - January 2-February 29, 2020. MMWR Morb. Mortal. Wkly. Rep. 69 (11), 307–311. 10.15585/mmwr.mm6911e1 32191691 PMC7739977

[B13] Pfefferle S. Reucher S. Norz D. Lutgehetmann M. (2020). Evaluation of a quantitative RT-PCR assay for the detection of the emerging coronavirus SARS-CoV-2 using a high throughput system. Euro. Surveill. 25 (9), 2000152. 10.2807/1560-7917.ES.2020.25.9.2000152 PMC7068162 32156329

[B14] Rabaan A. A. Al-Ahmed S. H. Haque S. Sah R. Tiwari R. Malik Y. S. (2020). SARS-CoV-2, SARS-CoV, and MERS-COV: A comparative overview. Infez Med. 28 (2), 174–184. 32275259

[B15] Sen S. Anand K. B. Karade S. Gupta R. M. (2020). Coronaviruses: origin and evolution. Med. J. Armed Forces India. 76 (2), 136–141. 10.1016/j.mjafi.2020.04.008 PMC7183968 32341622

[B16] Wang C. Horby P. W. Hayden F. G. Gao G. F. (2020). A novel coronavirus outbreak of global health concern. Lancet 395 (10223), 470–473. 10.1016/S0140-6736(20)30185-9 31986257 PMC7135038

[B17] Wang J. Cai K. He X. Shen X. Wang J. Liu J. (2020). Multiple-centre clinical evaluation of an ultrafast single-tube assay for SARS-CoV-2 RNA. Clin. Microbiol. Infect. 26 (8), 1076–1081. 10.1016/j.cmi.2020.05.007 32422410 PMC7227500

[B18] Wang W. Wang C. Bai Y. Zhang P. Yao S. Liu J. (2020). Establishment of reverse transcription recombinase-aided amplification-lateral-flow dipstick and real-time fluorescence-based reverse transcription recombinase-aided amplification methods for detection of the Newcastle disease virus in chickens. Poult. Sci. 99 (7), 3393–3401. 10.1016/j.psj.2020.03.018 32616233 PMC7597694

[B19] Wang X. Zhong M. Liu Y. Ma P. Dang L. Meng Q. (2020). Rapid and Sensitive Detection of COVID-19 Using CRISPR/Cas12a-based Detection with Naked Eye Readout, CRISPR/Cas12a-NER. Sci. Bull. (Beijing) 65 (17), 1436–1439 10.1016/j.scib.2020.04.041 32373393 PMC7198415

[B20] Wang Y. Cui Y. Yu Z. Li Y. Bai C. Sun P. (2020). Development of a recombinase-aided amplification assay for detection of orf virus. J. Virol. Methods 280, 113861. 10.1016/j.jviromet.2020.113861 32343981

[B21] Wu D. Wu T. Liu Q. Yang Z. (2020). The SARS-CoV-2 outbreak: What we know. Int. J. Infect. Dis. 94, 44–48. 10.1016/j.ijid.2020.03.004 32171952 PMC7102543

[B22] Xia S. Chen X. (2020). Single-copy sensitive, field-deployable, and simultaneous dual-gene detection of SARS-CoV-2 RNA via modified RT-RPA. Cell Discovery 6, 37. 10.1038/s41421-020-0175-x 32528725 PMC7253471

[B23] Xie M. Chen Q. (2020). Insight into 2019 novel coronavirus - An updated interim review and lessons from SARS-CoV and MERS-CoV. Int. J. Infect. Dis. 94, 119–124. 10.1016/j.ijid.2020.03.071 32247050 PMC7118633

[B24] Xiong Y. Luo Y. Li H. Wu W. Ruan X. Mu X. (2020). Rapid visual detection of dengue virus by combining reverse transcription recombinase-aided amplification with lateral-flow dipstick assay. Int. J. Infect. Dis. 95, 406–412. 10.1016/j.ijid.2020.03.075 32272263

[B25] Xue G. Li S. Zhang W. Du B. Cui J. Yan C. (2020). Reverse-Transcription Recombinase-Aided Amplification Assay for Rapid Detection of the 2019 Novel Coronavirus (SARS-CoV-2). Anal. Chem. 92 (14), 9699–9705. 10.1021/acs.analchem.0c01032 32441935

[B26] Yan C. Cui J. Huang L. Du B. Chen L. Xue G. (2020). Rapid and visual detection of 2019 novel coronavirus (SARS-CoV-2) by a reverse transcription loop-mediated isothermal amplification assay. Clin. Microbiol. Infect. 26 (6), 773–779. 10.1016/j.cmi.2020.04.001 32276116 PMC7144850

[B27] Zhang X. Guo L. Ma R. Cong L. Wu Z. Wei Y. (2017). Rapid detection of Salmonella with Recombinase Aided Amplification. J. Microbiol. Methods 139, 202–204. 10.1016/j.mimet.2017.06.011 28619662

